# A Genome-Wide mRNA Expression Profile in *Caenorhabditis elegans* under Prolonged Exposure to 1750MHz Radiofrequency Fields

**DOI:** 10.1371/journal.pone.0147273

**Published:** 2016-01-26

**Authors:** Yan Gao, Yiming Lu, Jianming Yi, Zhihui Li, Dawen Gao, Zhoulong Yu, Tongning Wu, Chenggang Zhang

**Affiliations:** 1 Beijing Institute of Radiation Medicine, Cognitive and Mental Health Research Center, State Key Laboratory of Proteomics, State Key Laboratory of Millimeter Wave, Beijing, 100850, China; 2 China Academy of Telecommunication Research of Ministry of Industry and Information Technology, No. 52 Huayuanbei Road, Beijing, 100191, China; Inserm U869, FRANCE

## Abstract

**Objective:**

*C*. *elegans* has been used as a biomonitor for microwave-induced stress. However, the RF (radiofrequency) fields that have been used in previous studies were weak (≤1.8W/kg), and the bio-effects on *C*. *elegans* were mostly negative or ambiguous. Therefore, this study used more intense RF fields (SAR = 3W/kg) and longer time course of exposure (60h at 25°C, L1 stage through adult stage) to investigate the biological consequences of 1750 MHz RF fields in wild-type worms.

**Methods:**

The growth rates and lifespans of RF-exposure group and the control group were carefully recorded. RNA samples were collected at L4 (35h) and gravid adult (50h) stages for further high-throughput sequencing, focusing on differences between the RF-exposure and the sham control groups.

**Results:**

The RF-exposed and sham control groups developed at almost the same rate and had similar longevity curves. In L4 stage worms, 94 up-regulated and 17 down-regulated genes were identified, while 186 up-regulated and 3 down-regulated genes were identified in adult stage worms. GO analysis showed that the differentially expressed genes at 35h were associated with growth, body morphogenesis and collagen and cuticle-based development. Genes that were linked to growth rate and reproductive development were differentially expressed at 50h. Some embryonic and larval development genes in the offspring were also differentially expressed at 50h. Ten genes were differentially expressed at both 35h and 50h, most of which were involved in both embryonic and larval developmental processes. Although prolonged RF fields did not induce significant temperature increase in RF exposure groups, the temperature inside worms during exposure was unknown.

**Conclusions:**

No harmful effects were observed in prolonged exposure to 1750 MHz RF fields at SAR of 3W/kg on development and longevity of *C*. *elegans*. Although some differentially expressed genes were found after prolonged RF exposure, these differences were ascribed to oscillating gene expression patterns in L4 and gravid adult worms. It was also difficult to rule out a weak thermal effect caused by prolonged RF exposure inside the worms.

## Introduction

With the rapid development of electric power and wireless communication technologies over the past several decades, public concerns have been raised about the possible health impact of exposure to occupational and environmental electromagnetic fields (EMF). Epidemiological data suggests that exposure to EMF may be associated with an elevated risk of cancer and other diseases in humans [1]. The International Agency for Research on Cancer (IARC) has classified electromagnetic fields of extremely low frequency (ELF) and RF as possible group 2B carcinogens [2, 3]. Laboratory research, however, has given no consistent evidence that EMFs of the magnitudes that are encountered in everyday life and over a substantial period of time can affect biological processes, nor is there consistent evidence that EMF affects the risk of cancer in animals.

Numerous studies have been conducted to elucidate the molecular mechanisms that mediate the possible effects of RF fields on cells or organisms[4–6], but the molecular targets are not yet known., Considering that biological effects of EMFs depended on sensitivity of experimental model and selecting of exposure parameters, conclusions from different studies were usually incomparable. For example, EMFs at a low dosage had less obvious biological effects in cultured cells[7–9], while EMFs athigh dosage caused typical heat stress [10, 11]. Therefore, the issue of whether there are health hazards associated with EMFs remains controversial. Thus, new research strategies must be sought.

The nematode *C*. *elegans* is the first multicellular organism whose genome has been completely sequenced [12, 13]. Its small size, transparency and short life cycle make it an informative model for studying aging, genetics, and whole-body stress[14, 15]. It is generally believed that there are two different types of biological effects of microwave radiation (including mobile phone radiation): thermal effects and non-thermal effects, which have been investigated in the *C*. *elegans* model in several studies [16–18]. The transgenic *hsp16-*GFP-*lac*Z *C*. *elegans* strain has often been used as a biomonitor of pollution[19] or microwave-induced stress[20]. Prolonged exposure to weak microwave fields has been reported to induce a heat-shock response in *C*. *elegans* [18]. The RF fields that were used in previous studies were much weaker (≤1.8W/kg) and the bio-effects were mostly negative or ambiguous. The *C*. *elegans* model was affected not only by the intensity of and the duration of exposure to RF fields but also by the accuracy of the exposure apparatus and temperature control. Thus, this study aimed to investigate whether RF fields at high specific absorption rate (SAR) and long exposures can affect growth and gene expression in *C*.*elegans* under the precise temperature control of the sXc-1800 exposure system. Considering that worms were sensitive to a variety of exogenous stressors at 25–26 ^0^C [21, 22], 25°C was chosen for use during microwave RF exposure. High-throughput sequencing was performed at two different time points, 35h (L4) and 50h (gravid adult stage), to reduce the influence of gene oscillations that occur during changes in the larval stages. The gene expression profiles were compared between RF exposure and sham control groups. In addition, the overlapping genes that were differentially expressed at both L4 and gravid adult stages were also analyzed. This study investigated the effects of high intensity and long exposure RF fields to address the mechanism of response in *C*.*elegans* to electromagnetic radiation.

## Materials and Methods

### Worm Culture

The wild-type strain N2 was obtained from the *Caenorhabditis* Genetics Center (CGC) and was grown on nematode growth medium (NGM) agar plates, as described by Sulston[23]. Gravid animals were collected and treated with a hypochlorite/NaOH solution to isolate eggs, which were incubated in M9-buffer solution without food for 16 h at 20°C to hatch L1 larvae. Synchronized L1 larvae were placed on 30mm NGM agar plates that were seeded with *Escherichia coli* OP50. An ambient temperatures of 25°C (±0.1°C) was used both routine exposure and sham control conditions. Worms in the two groups were drawn from the same source population.

### Microwave Exposure System

The sXc-1800 exposure system (IT’IS Foundation, Zurich, Switzerland) was utilized in the experiments. This system is comprised of two waveguides that are installed in an incubator. During exposure, one waveguide is randomly excited while the other acts as the sham control. In each waveguide, six 3.5-inch Petri dishes with NGM were placed inside the incubator. The SAR of the cultures could be set by inputting the dielectric properties of the culture (conductivity was 1.81 and relative permittivity was 74.51 for 1750 MHz). The temperature rise in the waveguide was monitored by a temperature sensor. As such, the real-time temperature rise could be reflected by the console software with an accuracy of ±0.1°C. During the experiments, the worms were held at 25 ^0^C, and worms in stages L1 through adult stage (0~60h after hatching) were exposed to CW signals of RF fields. The average SAR that was displayed in the worms was 3W/kg. In a similar exposure experiment, Dawe *et al*. [16] proposed a SAR gradient along the z-direction (from the dorsal to the ventral body wall) that was about 4W/kg/mm. We therefore concluded that the mean exposure dose for *C*. *elegant* was 3.2 W/kg at the ventral wall and 2.8 W/kg at the dorsal wall. The recorded temperature increase was less than 0.05°C in the experiment. The uncertainty for the exposure experiment was 19.28% according to the console software.

### Body Size and Growth Rate Analysis

More than 30 worms in each group were randomly photographed using a Nikon SMZ-1500 dissecting microscope set at 30x magnification and NIS-Elements F 4.0 software. The parameter that was used for body size analysis was pixels of each worm, as measured by Image-Pro Plus version 5.1 software (Media cybernetics). For growth rate analyses, synchronized larvae were initially measured every three hours from 20h to 60 h after the L1 stage (time zero). The growth curve of each group was created using Origin version 8.5 software.

### Analysis of Lifespan

One hundred age-synchronized worms in each group were used for lifespan analysis. From the young adult stage, animals in each group were transferred to new plates every few days until all of the worms were dead. Animals were assessed every 2 days and were scored as dead if pharyngeal pumping ceased and if the worms no longer responded to gentle prodding with a platinum wire. Animals that died from bagging or ruptured vulva or that crawled off the plate were excluded. Statistical analyses were performed on the RF field group versus the sham control group.

### RNA Sample Preparation for High-Throughput Sequencing

Four different samples were collected for high-throughput sequencing, including RF field exposure 35h (sample A), sham control 35h (sample B), RF field exposure 50h (sample C) and sham control 50h (sample D). According to the morphological features [24], worms in samples A and B were at the L4 stage, while worms in samples C and D were at the gravid stage. For RNA sample preparation, worms were picked and washed three times with M9 buffer to remove OP50, then were pelleted by centrifugation (274xg for 1min) and dropped in small concentrated pellets into liquid nitrogen, and finally stored at -80°C for RNA extraction. Total RNA was extracted using a Total RNA Kit I (Omega) following the manufacturer's instructions, was post immediately on dry ice and was sent to BGI (Beijing Genome Institute at Shenzhen) for High-throughput Sequencing (Illumina HiSeq 2000). To reduce the background noise, three independent exposure and sham control runs were taken. We compared six high throughput sequencings of pooled RNA populations from sham control L4 and gravid adult worms against six high throughput sequencings of pooled RNA from RF exposed L4 and gravid adult worms (taken from the same source population in each run).

### Data Analysis

*Caenorhabditis elegans* reference genome sequences (ce10,Oct, 2010) and the corresponding annotated RefSeq genes were downloaded from the UCSC Genome Brower [25] website (http://genome.ucsc.edu/). All of the raw data from RNA-seq were mapped to this reference genome using the TopHat [26] software, which was designed for mapping sequencing reads to a genome while considering the exon-exon junction information. Novel transcripts were removed from the analysis. The mapped data of three replicates of each group were inputted into the Cufflinks [27] software for gene expression difference test and averaged gene expression levels were reported in FPKM (fragments per kilobase of transcript per million fragments sequenced). We performed the Benjamini–Hochberg procedure of FDR analysis in gene expression difference tests to control the potential false positives introduced by multiple testing. The thresholds of false discovery rate (FDR) < 0.01 and absolute log fold change > 2 were used to determine the significantly upregulated and downregulated genes between different groups. Gene ontology analysis was implemented using David software[28] and a plugin of Cytoscape named BiNGO [29], which calculates overrepresented GO terms in the network and displays them as a network of significant GO terms.

### Statistical Analysis

Survival curves were estimated by a Kaplan-Meier function and the significance of difference between the curves was given by the Gehan-Wilcoxon test.

## Results

### The Development of *Caenorhabditis elegans* under 1750 MHz RF Fields

**Fig 1:**
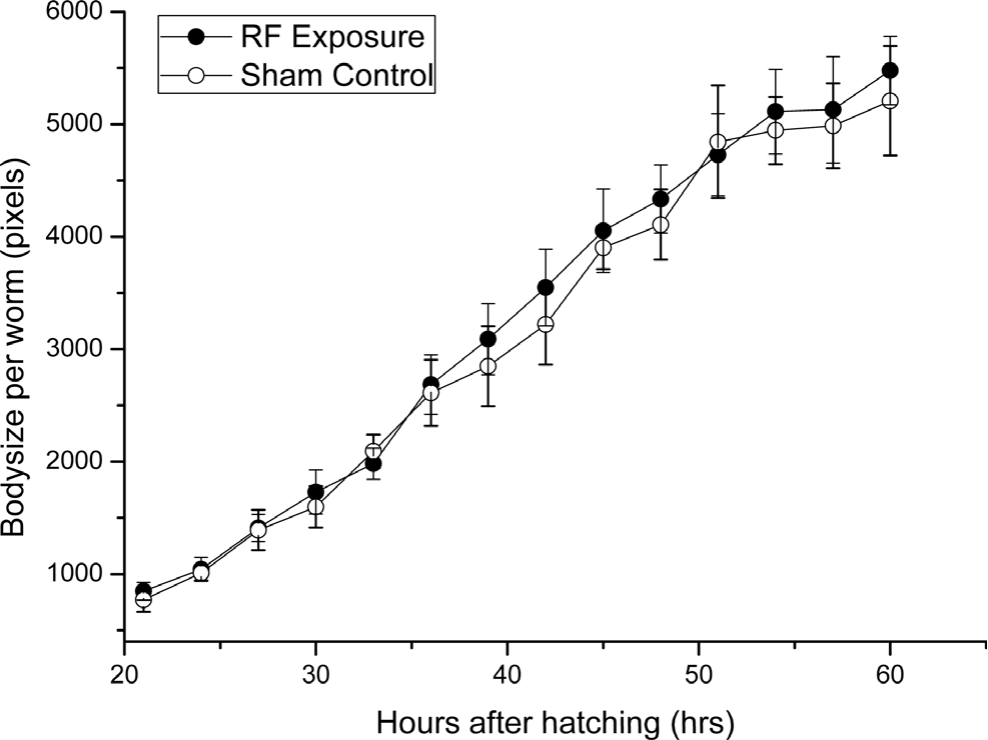
The growth curve of worms in RF exposure and sham control groups.

**Fig 2:**
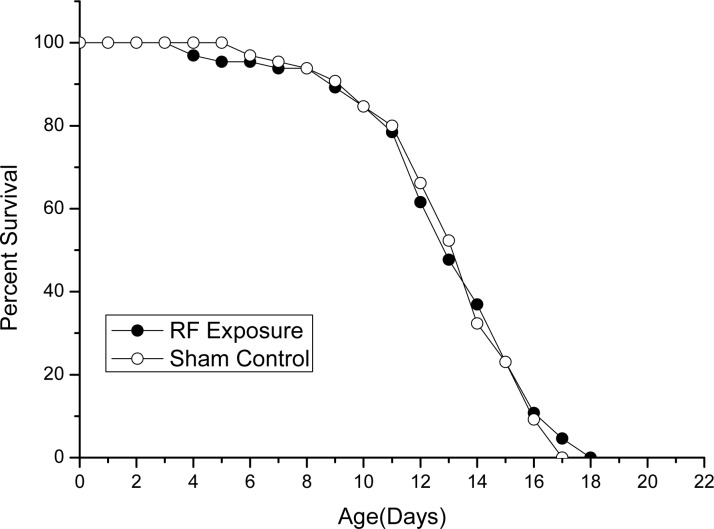
The longevity curve of RF exposure group and sham control groups. The significance levels (*P-*value) were analyzed between RF exposure and sham control group and were non-significant (*P* = 0.765).

The ambient temperature of 25°C (±0.1°C) was used in both exposure and sham control conditions. The body sizes of worms in exposure groups were not significantly different than those in the sham control groups at each time point (Fig 1). Accordingly, the longevity of worms that were subjected to RF fields exposure and that were subjected to sham exposure was almost identical (Fig 2) (*P* = 0.765). This implied that the worms in both groups developed at a similar rate. Based on a visual examination of the developmental age in the population, prolonged exposure to1750 MHz RF fields seemed not to influence development of *C*.*elegans*. Considering the close relationship between temperature and development rate of *C*.*elegans*, temperature change was monitored during experiment. The sXc-1800 exposure system has much greater confidence in the consistency and accuracy of temperature and SAR parameters, in addition, the real-time temperature rise could be reflected by the console software with an accuracy of ±0.1°C. The recorded temperature increase was less than 0.05°C, which meant the ambient temperatures inside the incubator of RF exposure group was within 0.05°C higher than sham control. However, the temperature changes inside worms was difficult to monitor during the RF exposure.

### Genome-Wide mRNA Alterations Are Induced by RF Field Exposure

**Fig 3:**
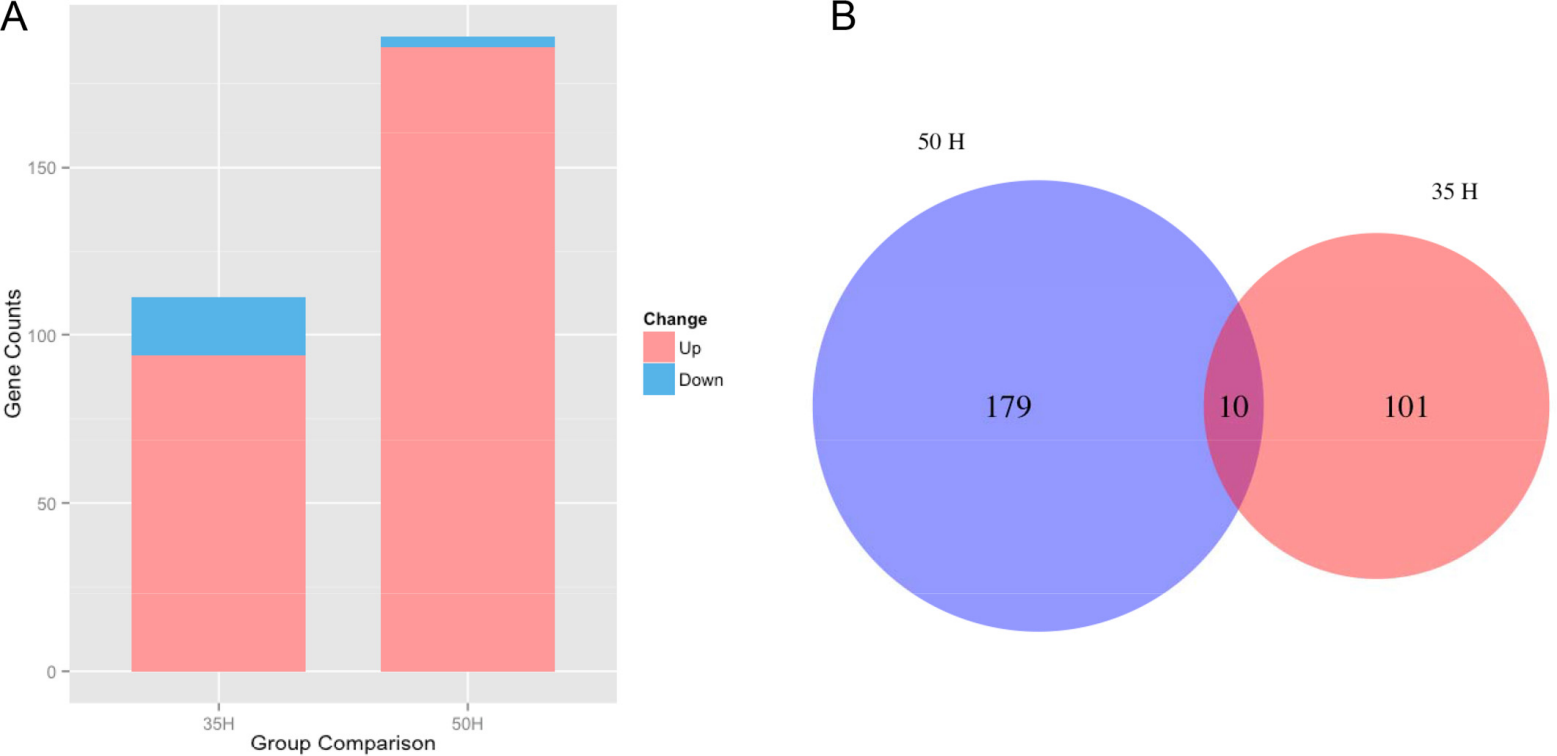
(A) Gene expression changes in two comparisons; (B) Venn diagram of differential genes in two comparisons. The significance levels (*P-*value) were analyzed between RF exposure and sham control groups. There were 94 upregulated and 17 downregulated genes at 35h and 186 upregulated and 3 downregulated genes at 50h. There were ten genes that were differentially expressed in both time points. The thresholds of false discovery rate (FDR) < 0.01 and absolute log fold change > 2 were used to determine the significantly upregulated and downregulated genes between different groups.

To answer the question of whether prolonged exposure to RF fields influences the gene expression profile, high-throughput sequencing was performed. The total mRNAs of each *C*. *elegans* sample (containing 22,150 genes) was completely sequenced using Illumina HiSeq 2000[30]. In total, over 230 million paired-end reads of 150 base pairs (bp) in length were acquired, and the total size of the reads was over 69 gigabases (Gb). The transcriptional level of mRNA was used to represent the expression level of the corresponding gene, and greater attention was given to the most meaningful comparisons of RF field exposure vs. sham control at L4 and gravid adult stage respectively. First, 94 upregulated and 17 downregulated genes were identified when comparing sample A and B (Fig 3A, S1 Table). Additional high-throughput sequencing was performed at a later time point that was well-separated from the oscillations that occur during changes in larval stages; at this stage, even more genes had altered expression in response to RF field exposure. There were 186 upregulated and 3 downregulated genes when comparing samples C and D (Fig 3A, S2 Table). There were ten genes that were differentially expressed in both the comparisons between treated and untreated L4 worms (35h) and that between treated and untreated gravid worms (50h) (Fig 3B).

### The Gene Ontology-Based Functional Analysis of Differentially Expressed Genes in Larval Stage 4

**Fig 4:**
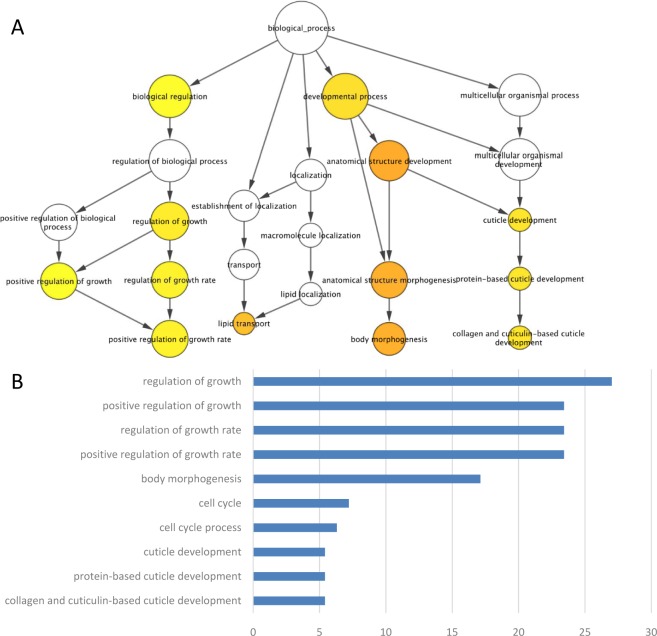
The Gene Ontology-based functional analysis of differentially expressed genes in larval stage 4. (A) The Gene Ontology-based functional analysis of differentially expressed genes in the form of biological network; (B) Top 10 enriched Gene Ontology terms of RF exposure associated genes. The significance levels (*P-*value) were analyzed between RF exposure and sham control groups. Only GO terms that were enriched > 1.5 fold over the average and with a *P*-value < 0.05 were considered to be significant enriched terms.

A Gene Ontology (GO) [31] analysis was then performed for the genes whose expression was altered at L4 stage under RF field exposure. Only GO terms that were enriched > 1.5 fold over the average and with a *P*-value < 0.05 were considered to be significant enriched terms. Several RF field induced genes at 35h were mapped to pathways that were associated with growth regulation, development processes and cuticle development (Fig 4A). The most enriched GO terms also showed that genes that were linked to growth regulation (*P* = 0.0011), body morphogenesis (*P* = 1.38E^-6^), and collagen and cuticulin-based development (*P* = 0.001) were differentially expressed when comparing groups comparison A and B (Fig 4B, S3 Table). It is worth mentioning that worms were at transformational stage from L4 to young adult at 35h after hatching and with the onset of molting and reproductive development according to their morphology. Thus, some of the upregulated genes have oscillation profiles during development; for example, cuticle development and molting associated genes (e.g. *dpy*, *col*, *sqt*, *lon*, *rol*, *nrh-6*,*et* al.). Some growth regulation and reproductive development genes were upregulated in RF exposure group, for example, *emb-5*, *dao-4*, *dod-3*, *fkb-4*, *et* al. The results above illustrate that a prolonged exposure to RF fields may promote expression of development-associated genes in larval stage 4.

### The Gene Ontology-Based Functional Analysis of Differentially Expressed Genes in Gravid Adult Worms

**Fig 5:**
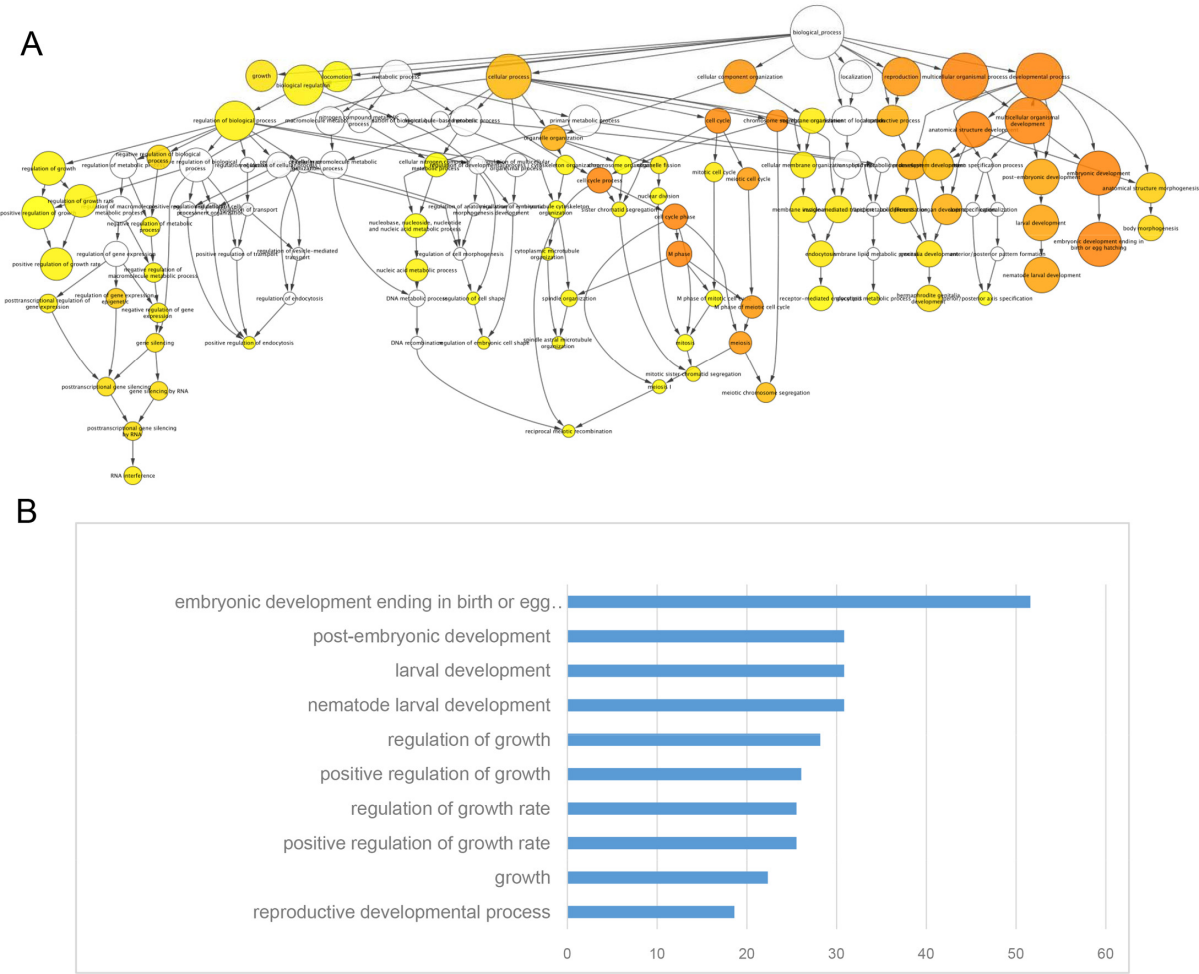
The Gene Ontology-based functional analysis of differentially expressed genes in gravid adult worms. (A) The Gene Ontology-based functional analysis of differentially expressed genes in the form of biological network; (B) Top 10 enriched Gene Ontology terms of RF exposure associated genes. The significance levels (*P-*value) were analyzed between RF exposure and sham control groups. Only GO terms that were enriched > 1.5 fold over the average and with a *P*-value < 0.05 were considered to be significant enriched terms.

A Gene Ontology (GO) analysis of the genes with differential expression at the adult stage in worms that were exposed to RF fields showed that many differently expressed genes can be mapped to embryonic, post-embryonic and nematode larval developmental processes (p< 0.01) (Fig 5A and 5B). This is likely due to the onset of egg laying in the gravid adult worms and larval development in the offspring. Several RF field-induced genes in the adult stage were also mapped to pathways that were associated with the regulation of the growth rate (Fig 5A). The most enriched GO terms also showed that genes that were linked to the positive regulation of the growth rate were differentially expressed when comparing comparison C and D (p< 0.01) (Fig 5B, S4 Table), which was similar to the L4 stage. In addition, reproductive development was also an important enriched GO term (p< 0.01).

### The Overlapping Genes between Two Comparisons and Gene Ontology-Based Functional Analysis

The expression profiles and function of overlapping genes are shown in Table 1. Most these overlapping genes were upregulated during RF field exposure. Half of these genes, including *rfp-1*, *pbrm-1*, *hoe-1*, *glp-1*and *emb-5*, express in several stages from embryonic to adult. In addition, these genes function in both reproductive developmental processes and positive growth rate regulation. Five other genes, including *npa-1*, *C32E8*.*11*, *T08G11*.*1*, *T19A5*.*1* and *Y66H1B*.*5*, function in molecular biological events in development progress, for example, lipid binding, ubiquitination and protein processing. These results showed that prolonged RF field exposure may promote development and growth rate-associated genes expression at both the L4 and gravid adult stages as well as in embryonic development. Although these effects were induced by RF exposure, it was still difficult to eliminate the possibility of an effect from subtle temperature changes inside worms.

**Table 1:** Genes Showing Significant Expression Changes in RF-Exposed Compared to Sham Controls at both L4 and adult gravid stages.

	genes	35h-Ctrl	35h-RF	P-Value*	50h-Ctrl	50h-RF	P-Value*	Overview of function^#^
1	*emb-5*	4.97	14.93	1.01E-08	5.92	51.07	3.25E-06	cell division (embryo); gonad development, downstream effector in Notch-like signaling pathways
2	*glp-1*	4.52	13.93	1.08E-08	7.14	49.68	3.05E-05	cell fate specification in germline and somatic tissues, LIN-12/Notch family
3	*hoe-1*	23.47	50.94	2.11E-07	13.5391	53.5761	1.29E-04	fertility, normal growth progression during the late larval stages and germ line proliferation
4	*pbrm-1*	4.81	11.19	2.19E-05	4.52	18.47	6.00E-05	function in chromatin remodeling and transcriptional regulation required for vulval development
5	*rfp-1*	4.98	10.83	9.02E-05	5.51	39.42	3.90E-07	inhibits physiological in oocytes apoptosis, required for vulval development, egg-laying, progression throughout L1
6	*npa-1*	27.44	86.56	3.35E-14	9.39	64.95	8.71E-08	lipid binding
7	*C32E8*.*11 (ubr-1)*	5.24	12.47	3.24E-06	2.93	17.91	1.19E-04	Ubiquitin conjugating enzyme binding and zinc ion binding activity
8	*T08G11*.*1*	3.73	7.60	3.08E-05	1.71	11.30	2.53E-05	An ortholog of human VPS13A and VPS13C
9	*T19A5*.*1*	4.61	10.29	6.21E-05	4.51	26.55	1.69E-04	an ortholog of human TTC17
10	*Y66H1B*.*5* (*fln-1*)	12.00	3.43	7.87E-07	1.54	9.83	1.25E-04	normal brood size
								ovulation

* The significance levels (*P-*value) were analyzed between control and RF exposure groups.

# Reference from http://www.wormbase.org/

## Discussion

*C*. *elegans* is an informative and convenient model for stress studies. It has been reported that prolonged exposure to weak RF fields can induce a heat-shock response in transgenic *C*. *elegans* strains (those which carry *lacZ* or GFP reporter genes that are under the control of heat-shock promoters–usually *hsp16*) [22]. The dosages of RF field exposure that were used in this study were higher (SAR of 3W/kg, 60h) than those that were routinely tested in many other published studies (SAR of 1mW/kg~1.8W/kg, up to 20h). The reporter gene *hsp16* in *C*. *elegans* is affected by very small differences in a small temperature range: there is no response at or below 27°C, while the maximal response in all worms that express galactosidase occurs at 30°C[32]. Because of the precise temperature control of the sXc-1800 exposure system (which has an accuracy of ±0.1°C), prolonged exposure to RF fields at 25°C induced temperature increase less than 0.05°C in RF exposure group. Therefore, the little temperature change did not influence development rate and longevity of worms in in RF exposure group. However, the temperature changes inside worms was unknown.

Physical and chemical factors in the environment have a strong impact on the development of *C*. *elegans*. For example, harsh environmental conditions, such as food shortage, high temperature or high population density, cause *C*.*elegans* larvae to arrest as stress-resistant “dauer” larvae after the L2 larva stage [33]. Therefore, prolonged RF field exposure, as a physical factor, may affect the development of nematodes. It is generally believed that lower temperatures extend the lifespan, while higher temperatures shorten it, and this has been shown in *C*. *elegans* [34]. High temperatures enhance the rates of chemical reactions, thereby accelerating the pace of aging. In this study, prolonged RF field exposure did not significantly affected the speed of worm development based on visual examination. However, the biological effects inside worms, for example, gene response profiles change, were unknown and needed to be assessed by high- throughput sequencings.

The results of high-throughput sequencing showed that the RF field that was used in this study significantly promoted gene expression in *C*.*elegans*. Considering the background noise of gene expression and statistical deviation, comparisons based on a single experiment cannot yield meaningful information on the consistency and robustness of gene expression changes [35]. Therefore, RF exposure and sham exposure were performed across several independent replicates in this study. In addition, gene expression differences at one time point were insufficient to reflect prolonged RF exposure bio-effects in worms. Therefore, gene expression profiles of two time points, covering L4 to adult stages, with three independent replicates were observed using high throughput sequencing. There were only 111 differentially expressed genes between sample A and B and 189 genes between C and D. According to the GO analysis, genes that were linked to growth regulation, body morphogenesis and collagen and cuticulin-based development were differentially expressed when comparing samples A and B (p< 0.01). Between samples C and D, genes that were linked to growth rate and to reproductive development were differentially expressed. Some embryonic and larval development genes in the offspring were also differentially expressed between these samples (p< 0.01). It seemed that prolonged RF field exposure promoted development associated genes in worms at both the L4 and gravid adult stages as well as in the offspring of gravid adult worms at 50h.

As *C*.*elegans* proceeds from the egg through four larval stages to the adult (egg laying) stage, the transition between the larval stages is marked by the synthesis of a new cuticle and the subsequent molting of the old one. There are many genes that demonstrated an oscillating multiphasic pattern during development. This was another reason for gene expression differences between the two groups. For example, collagen is a major component of the nematode’s extracellular cuticle, which is sloughed off and reformed at each of the four postembryonic molts under the direction of an underlying layer of hypodermal tissue[36, 37]. *C*.*elegans* experiences molting and reproductive development during the later L4 stage. The body morphogenesis genes and collagen and cuticulin-based developmental genes (the *dpy*, *col*, *sqt*, *lon*, *rol*, *nrh*, gene families) were upregulated in L4 worms at the transformational stage from L4 to young adult. In addition, genes that positively regulate growth rate, including *emb-5*, *dao-4*, *dod-3*, *fkb-4*, genes were also upregulated in RF field group. It was obvious that those genes that were upregulated in sample A were preparing for the transform stage from L4 to young adult.

The differences in the gene expression profiles when comparing sample groups C and D also reflected developmental phenotypes that were characteristic of adult phase, including mature reproductive organs and egg laying. For example, in addition to genes that positively regulated growth rate, embryonic development genes (*aco-2*, *lrs-1*, *rad-50*, *noah-1*, *etc*.), post-embryonic genes (*dcp-66*, *R07E5*.*1*, *ego-1*, *mrp-5*, *daf-16*, *daf-15*, *etc*.) and larval development genes (*aco-2*, *B0464*.*2*, *dcp-66*, *R07E5*.*1*, *ego-1*) were significantly up regulated after RF field exposure. This implied that prolonged RF field exposure promoted developmental gene expression in both adult worms and embryonic worms. Therefore, the overlap of two comparisons included genes that were expressed throughout embryonic to larval stages processed and genes that promoted reproductive developmental processes and the positive regulation of the growth rate. These also illustrated that prolonged RF exposure promoted larval and embryonic development at the genetic level.

It was interesting to find that *glp-1*, encoding a Notch-related receptor, was consistently altered by RF exposure at L4 (35h) and gravid adult (50h) stages in this study. Core components of Notch signaling pathway (e.g., GLP-1, LIN-12) have been conserved from worms to humans [38]. Proliferative germ cell divisions during larval development and GSC (germline stem cell) maintenance divisions in adults are controlled by the GLP-1/Notch signaling pathway [39]. It suggested that prolonged RF field exposure may influence germ cell divisions and reproductive organs function of worms. However, there were no significant differences in offspring numbers between the RF-exposure and the sham control groups (data not shown). Thus, prolonged RF field using in this study did not induce significant reproductive effects in worms. There is tremendous concern for the possible adverse effects of RF fields on reproductive system. Recent epidemiological studies[40], animal studies[41, 42] and in vitro laboratory studies[43] indicated the possible harmful effects of RF field on reproductive system. Other researchers reported that RF fields had no harmful effects on reproductive system [44, 45], therefore, there is still no clear consensus of opinion. This study may provide important references for reproductive effect study of electromagnetic fields.

Microwave radiation induces both thermal and non-thermal effects in biological systems. Worms that was exposure to RF fields and sham exposed controls were kept at the same ambient temperature and developed at almost the same rate. However, the actual internal temperature for these nematodes might be somewhat different than in the surrounding medium during RF exposure due to different dielectric properties. It was reported that even inside the worm body, dielectric properties were not uniform along the z-direction (from the dorsal to the ventral body wall)[16]. Although, it was difficult to monitor the temperature changes during exposure, the upregulated gene expression profiles in RF exposure groups illuminated that prolonged RF fields at SAR of 3W/kg may have a weak thermal effect inside worms, which significantly promote the developmental gene expression in *C*. *elegans*.

## Conclusion

No harmful effects were observed in prolonged exposure to 1750 MHz RF fields at SAR of 3W/kg on development and longevity of *C*. *elegans*. Although some differentially expressed genes were fund after prolonged RF exposure, these differences were ascribed to oscillating gene expression patterns in L4 and gravid adult worms. It was also difficult to rule out a weak thermal effect caused by prolonged RF exposure inside the worms.

## Supporting Information

S1 Table The significantly different expressed genes in L4 worms induced by RF field exposure. (XLSX)

S2 Table The significantly different expressed genes in adult worms induced by RF field exposure. (XLSX)

S3 Table The significantly enriched Gene Ontology terms of RF exposure associated genes in L4 worms. (XLSX)

S4 Table The significantly enriched Gene Ontology terms of RF exposure associated genes in adult worms. (XLSX)
